# Henry A. Riley, A. B., LL. B.

**Published:** 1892-07

**Authors:** 


HENRY A. RILEY, A. B., LL. B., counselor-at-law, died in New York, June 9, 1892, of heart disease, from which he had long been a sufferer. He has been a contributor to the JOURNAL, and his articles on medico-legal subjects are well known throughout the United States. The *New York Medical Journal* says: "Though a lawyer, he was well known to the medical profession by his contributions to the medical jurisprudence to this and other journals." For two years past he has been compelled to abandon his professional pursuits by reason of his ill-health, and he has devoted his time largely to writing upon medico-legal questions. He developed a special field that had heretofore been unoccupied, which was of service to the medical, as well as the legal, profession in the line that he pursued.

